# Supplementary material for: Prevalence of hypertension and its determinants in Ethiopia: A systematic review and meta-analysis
Source: PLoS One. 2020 Dec 31;15(12):e0244642. doi: 10.1371/journal.pone.0244642 (PMC7774863; doi:10.1371/journal.pone.0244642)
Supplement: S1 Table — (DOCX) [file pone.0244642.s001.docx]

| PubMed/MEDLINE database | | | | |
| --- | --- | --- | --- | --- |
| MeSH Heading | Entry term (Synonyms) | Combination | Articles | Date of search |
| Hypertension | Blood Pressure, High  Blood Pressures, High  High Blood Pressure  High Blood Pressures | (Hypertension[Title/Abstract] OR "Blood Pressure,  High"[Title/Abstract] OR "High Blood Pressure"[Title/Abstract] OR "High Blood Pressures"[Title/Abstract] OR "Blood Pressure, High"[Title/Abstract] OR "Blood Pressures, High"[Title/Abstract])AND Ethiopia[Title/Abstract]) | 192 | 24/10/2019 |
| Google scholar database | | | | |
| Hypertension | Blood Pressure, High  Blood Pressures, High  High Blood Pressure  High Blood Pressures | allintitle: Ethiopia Hypertension OR "Blood Pressure, High " OR "Blood Pressures, High" OR "High Blood Pressure" OR "High Blood Pressures" | 98 | 22/10/2019 |
| Hinari database | | | | |
| Hypertension | Blood Pressure, High  Blood Pressures, High  High Blood Pressure  High Blood Pressures | ((TitleCombined:(Hypertension)) OR (TitleCombined:("Blood Pressure, High")) OR (TitleCombined:("Blood Pressures, High")) OR (TitleCombined:("High Blood Pressure")) OR (TitleCombined:("High Blood Pressures"))) AND (TitleCombined:(Ethiopia)) | 151 | 03/11/2019 |
|  |  | ((Abstract:(Hypertension)) OR (Abstract:("Blood Pressure, High")) OR (Abstract:("Blood pressures, High")) OR (Abstract:("High Blood Pressure")) OR (Abstract:("High Blood Pressures"))) AND (Abstract:(Ethiopia)) | 339 | 03/11/2019 |
|  | Grey literature search |  | 2 |  |

# **S1 Table: Studies search strategies and entry terms from different electronic databases on the prevalence and determinants of hypertension**
