# Supplementary figures and images for: Prevalence of hypertension and its determinants in Ethiopia: A systematic review and meta-analysis
Source: PLoS One. 2020 Dec 31;15(12):e0244642. doi: 10.1371/journal.pone.0244642 (PMC7774863; doi:10.1371/journal.pone.0244642)

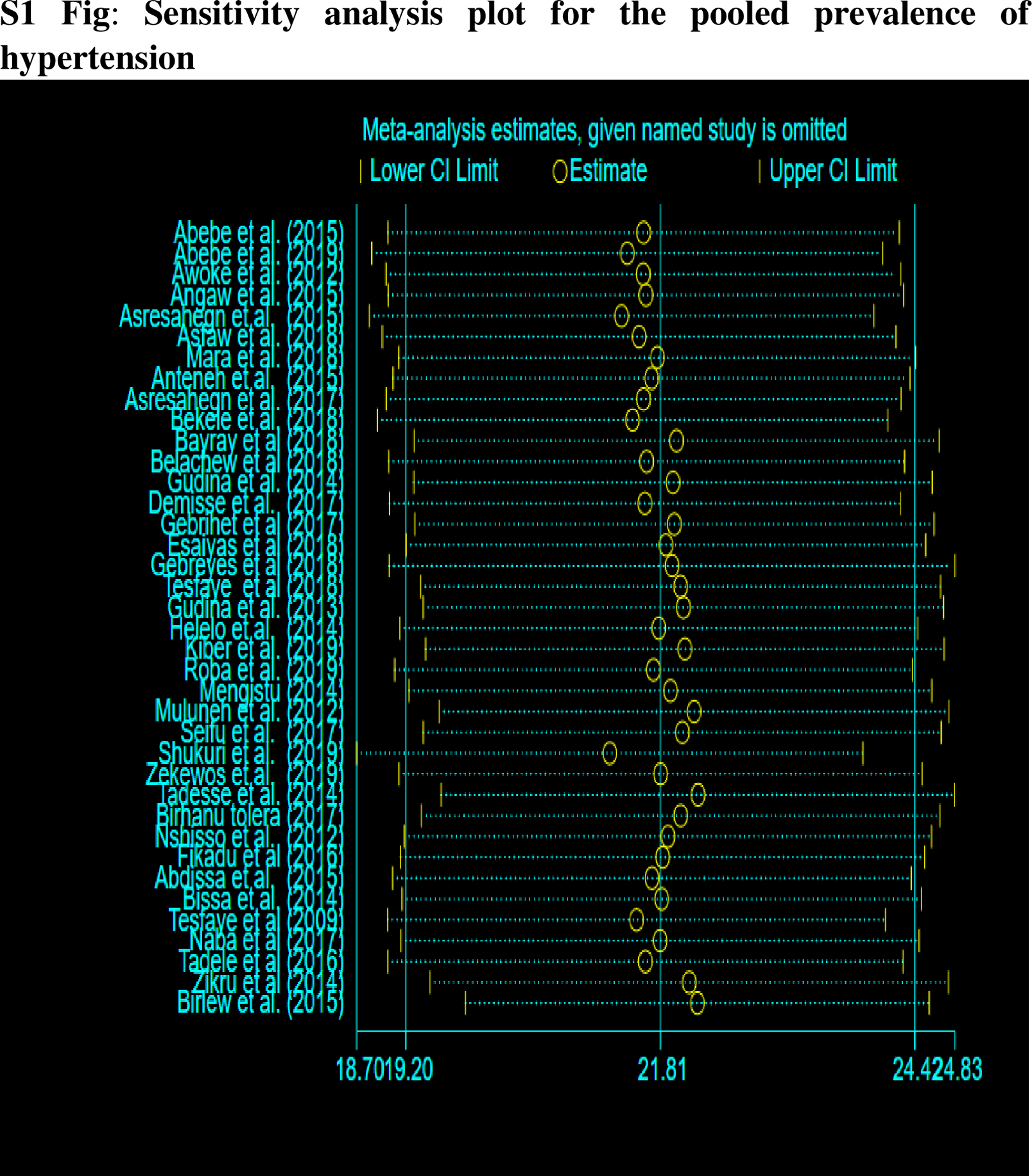

Supplement: S1 Fig — (TIF) [file pone.0244642.s002.tif]

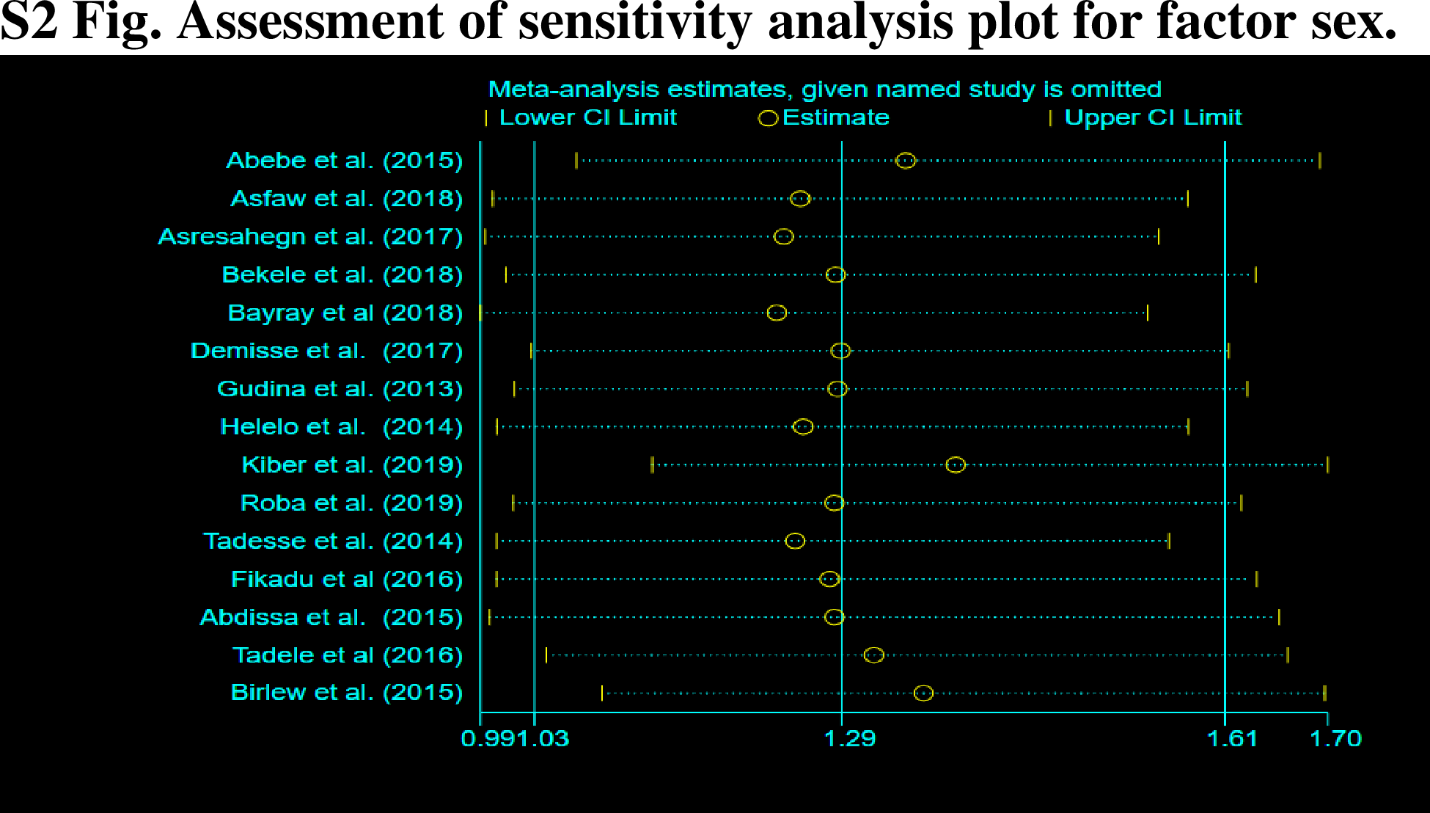

Supplement: S2 Fig — (TIF) [file pone.0244642.s003.tif]

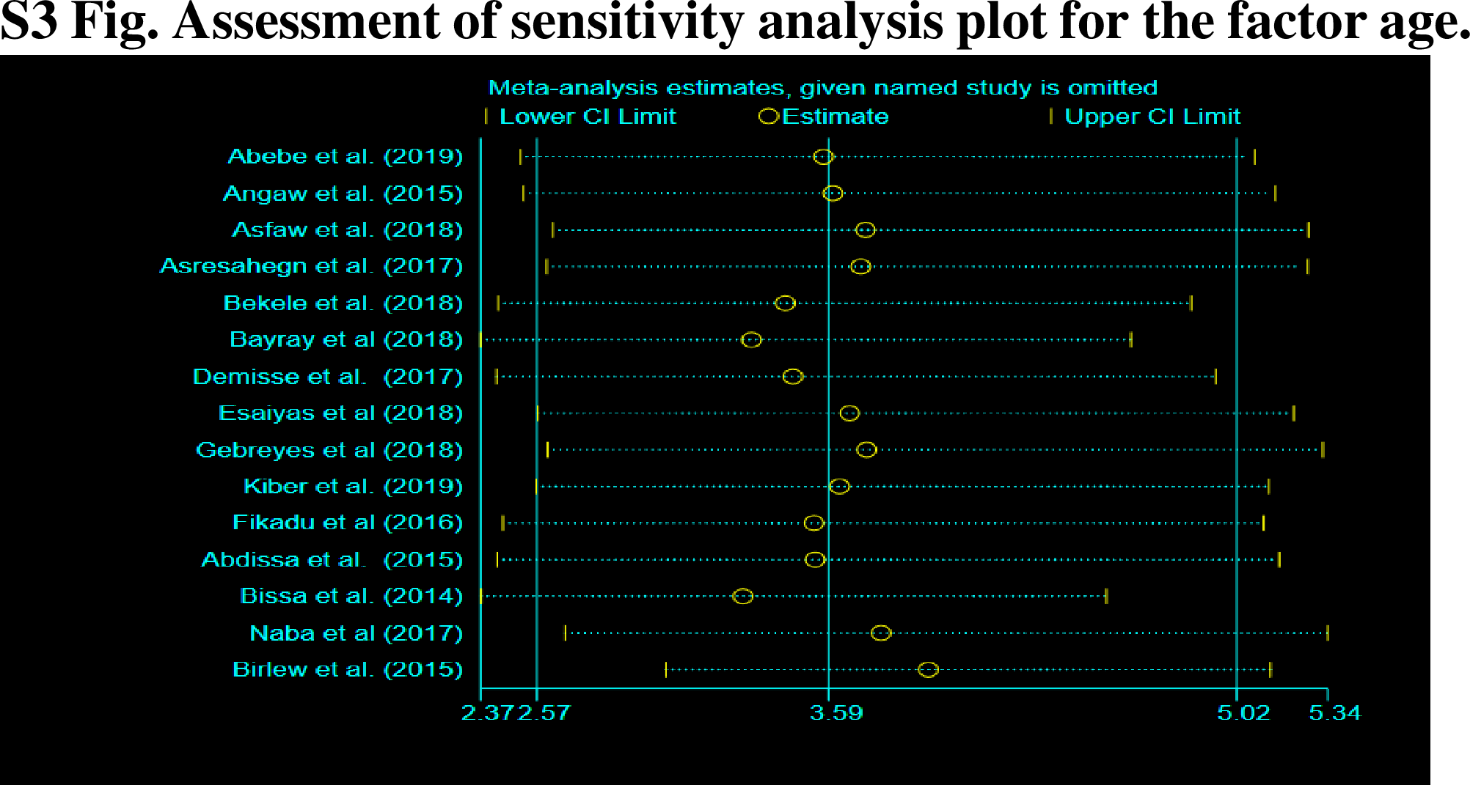

Supplement: S3 Fig — (TIF) [file pone.0244642.s004.tif]

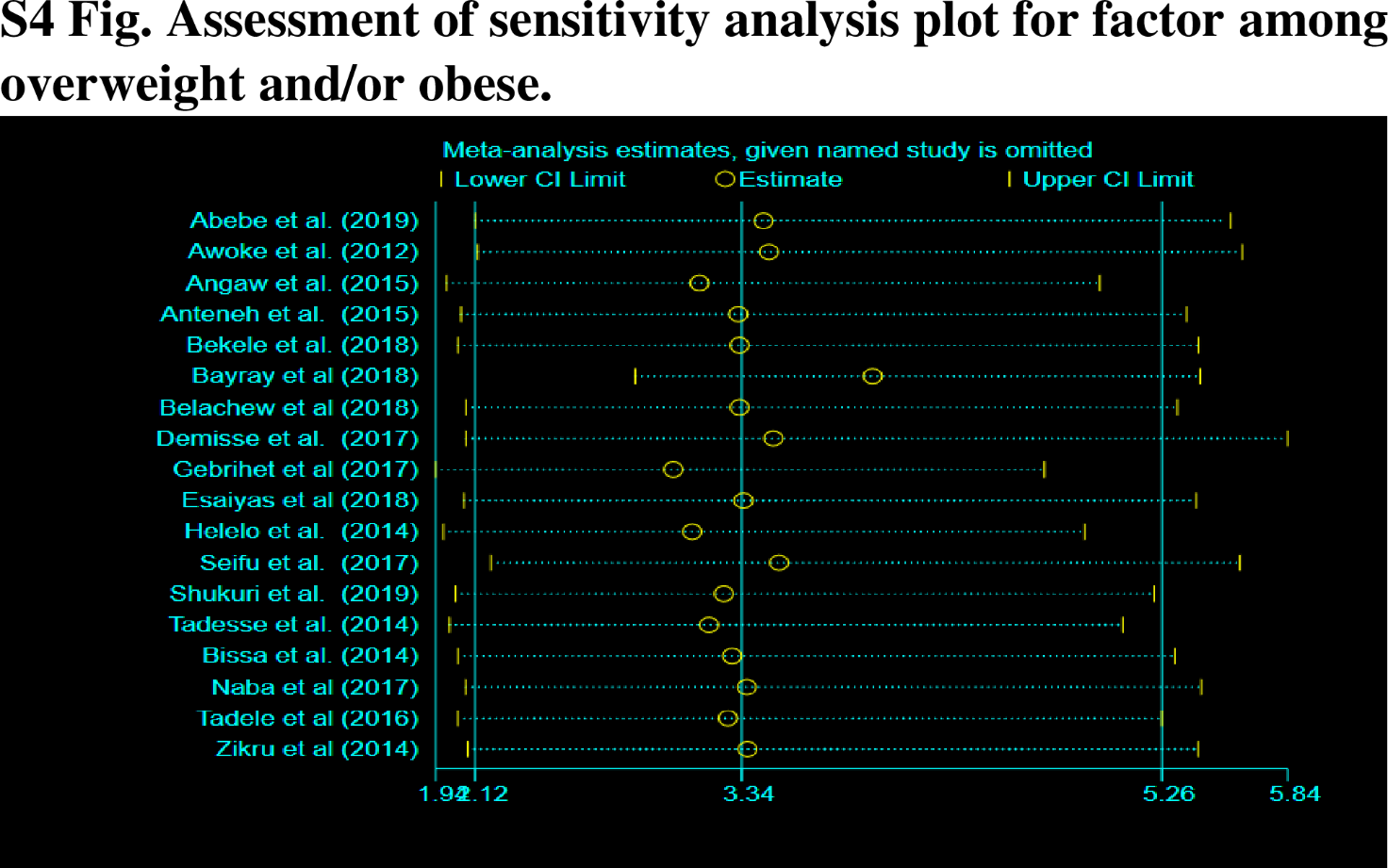

Supplement: S4 Fig — (TIF) [file pone.0244642.s005.tif]

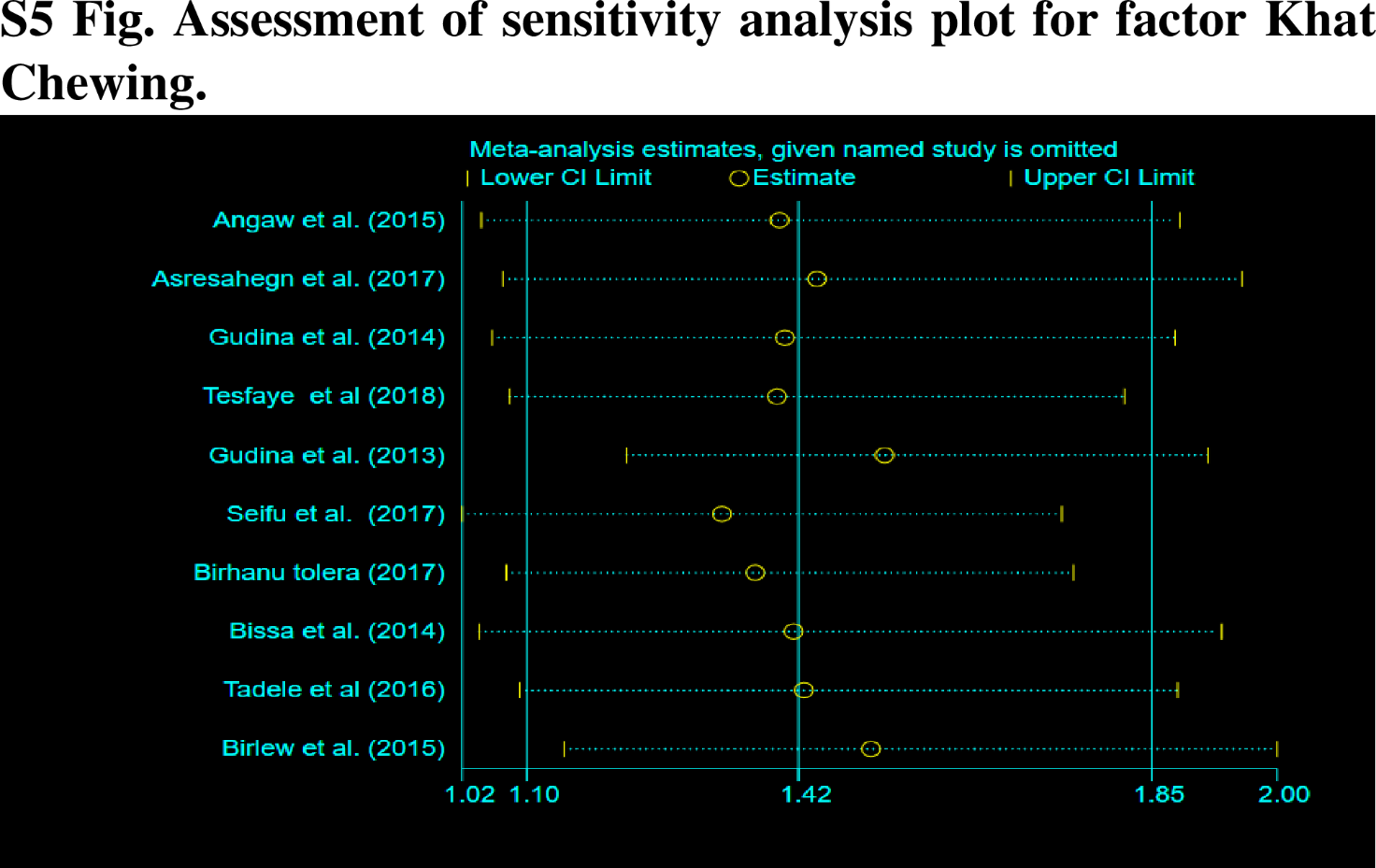

Supplement: S5 Fig — (TIF) [file pone.0244642.s006.tif]

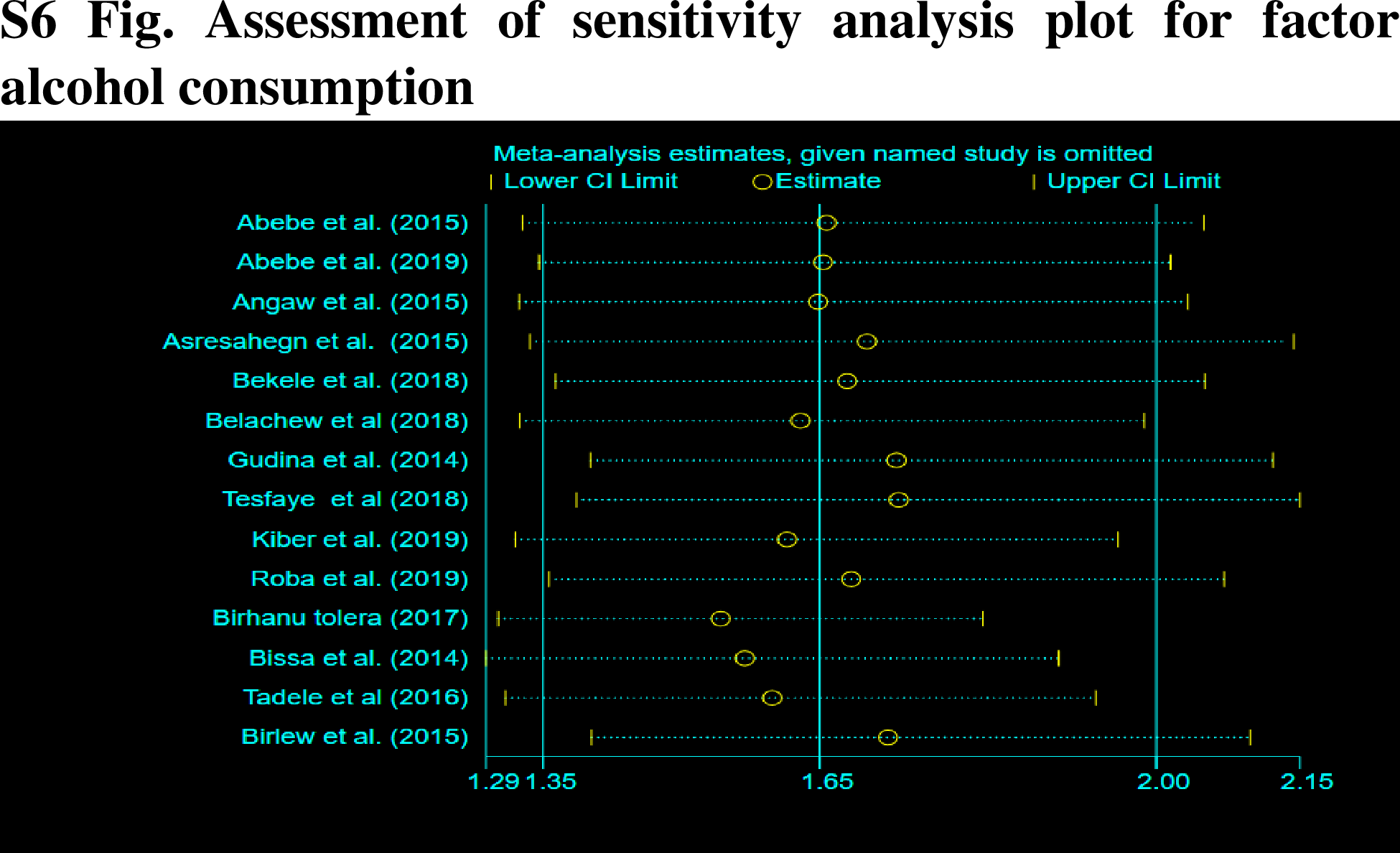

Supplement: S6 Fig — (TIF) [file pone.0244642.s007.tif]

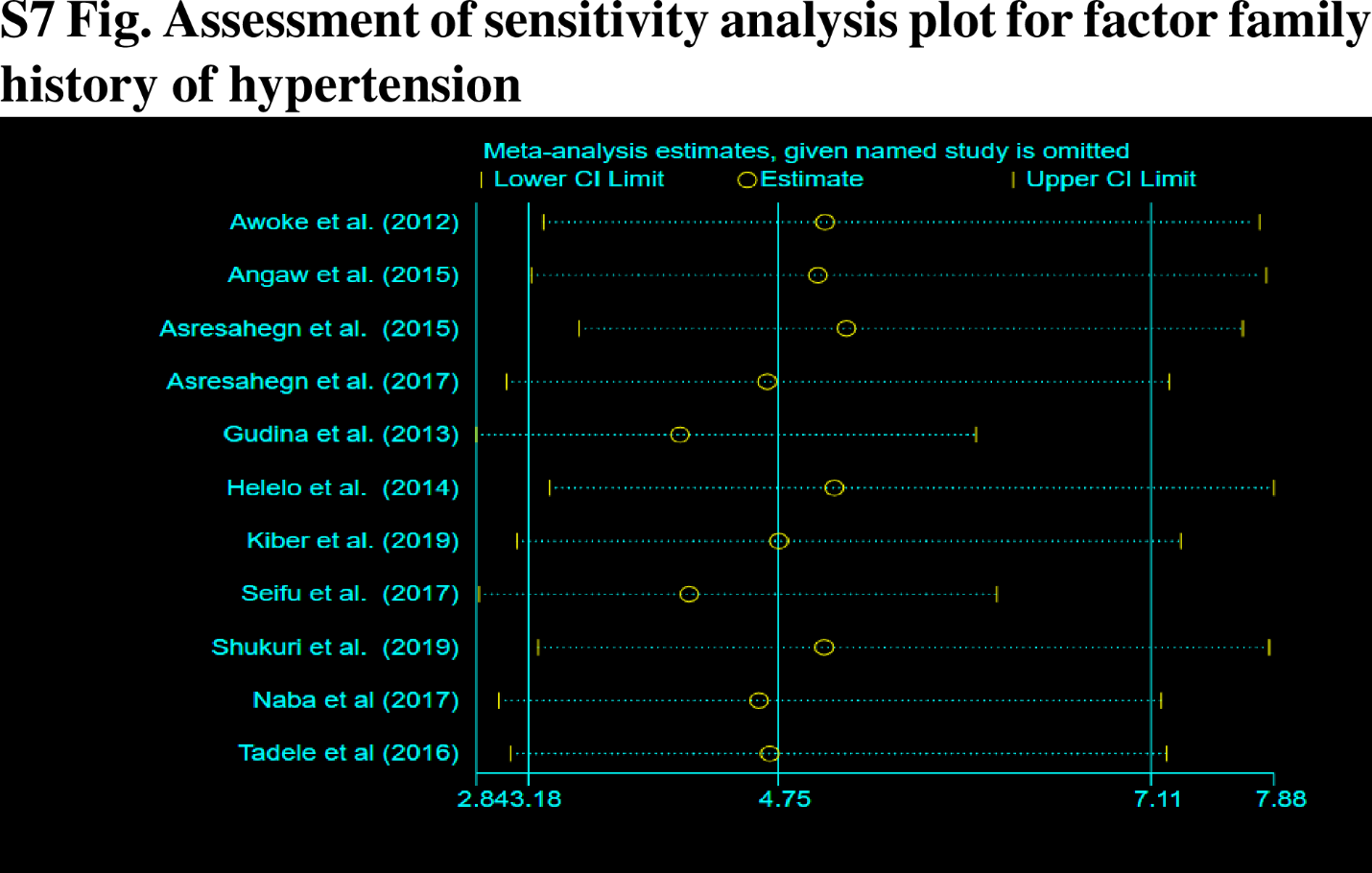

Supplement: S7 Fig — (TIF) [file pone.0244642.s008.tif]

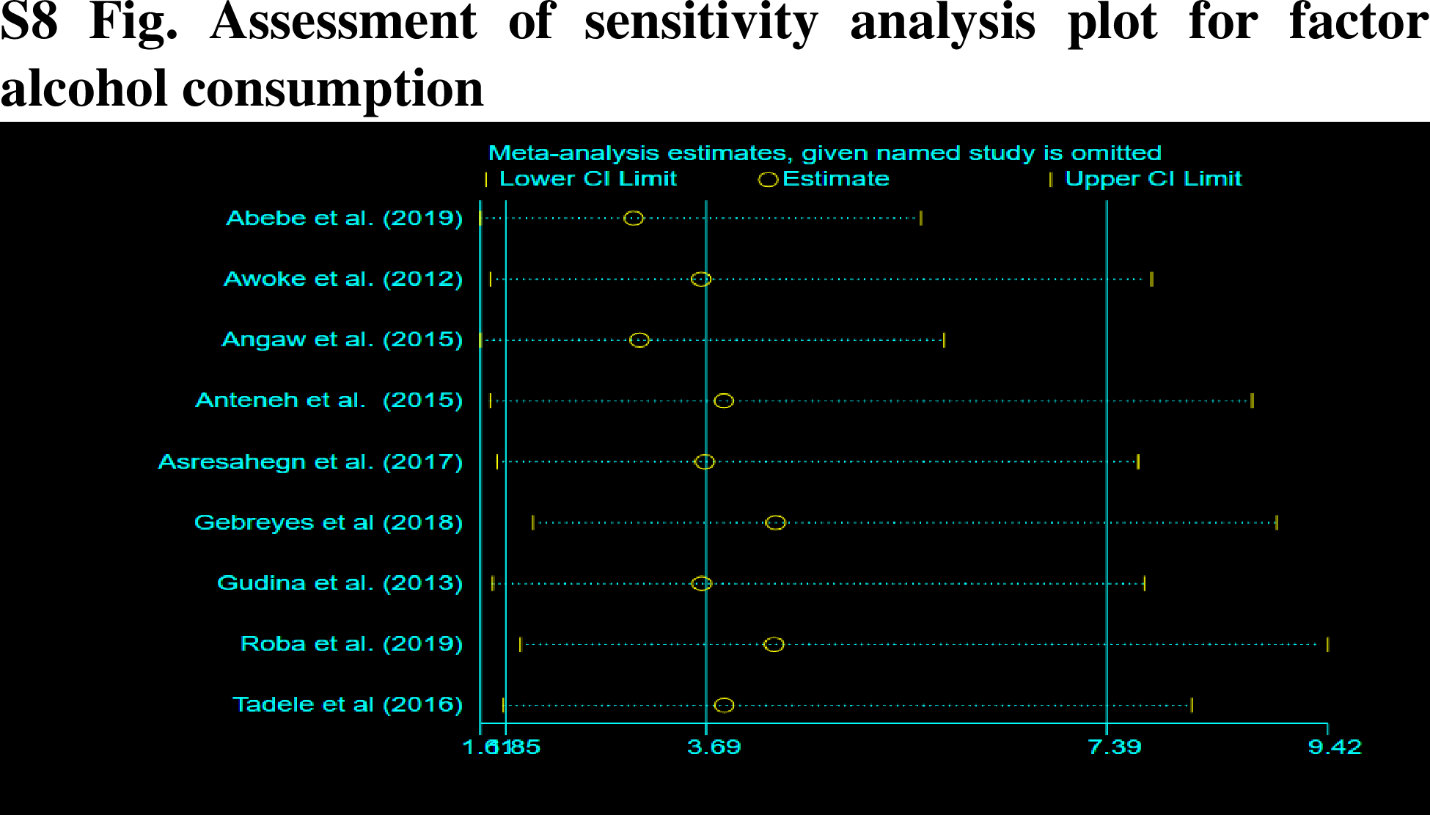

Supplement: S8 Fig — (TIF) [file pone.0244642.s009.tif]
