# Supplementary material for: Prevalence of hypertension and its determinants in Ethiopia: A systematic review and meta-analysis
Source: PLoS One. 2020 Dec 31;15(12):e0244642. doi: 10.1371/journal.pone.0244642 (PMC7774863; doi:10.1371/journal.pone.0244642)
Supplement: S1 Checklist — (DOC) [file pone.0244642.s010.doc]

| **Section/topic** | **#** | **Checklist item** | **Reported on page #** |
| --- | --- | --- | --- |
| **TITLE** | | |  |
| Title | 1 | Both systematic review and meta-analysis and meta-regression | 1 |
| **ABSTRACT** | | |  |
| Structured summary | 2 | Background; objectives; data sources; study eligibility criteria, participants, methods; results; conclusions and implications of key findings | 2 |
| **INTRODUCTION** | | |  |
| Rationale | 3 | Describe the rationale for the review in the context of what is already known. | 3 |
| Objectives | 4 | Provide an explicit statement of questions being addressed with reference to participants, interventions, comparisons, outcomes, and study design (PICOS). |  |
| **METHODS** | | |  |
| Protocol and registration | 5 | Registered before three month and waiting from PROSPERO registration number. | Up on request |
| Eligibility criteria | 6 | CoCoPop (Condition, Context, Population) approach for prevalence studies to declare inclusion and exclusion criteria. | 4 |
| Information sources | 7 | Studies were identified using databases PubMed/MEDLINE, Hinari, Google scholar, and google search. The date of search was performed on 10-11/24/2019 from all databases. | 4 |
| Search | 8 | The searching combination used as; Hypertension OR "Blood Pressure, High” OR "High Blood Pressure" OR "High Blood Pressures" OR "Blood Pressure, High" OR "Blood Pressures, High" | 4 |
| Study selection | 9 | Screening of articles based on Title, Abstract and full text review. | 5 |
| Data collection process | 10 | Through review from title, abstract and full text. | 5 |
| Data items | 11 | List and define all variables for which data were sought (e.g., PICOS, funding sources) and any assumptions and simplifications made. |  |
| Risk of bias in individual studies | 12 | The quality of included studies were assessed using the modified version of a quality assessment tool for prevalence studies which is validated in the previous study | 5 |
| Summary measures | 13 | Summary measures Odds Ratio (OR) and Coefficients. | 6 |
| Synthesis of results | 14 | Pooled analysis, Sensitivity analysis, Subgroup analysis, meta regression, testing publication bias were performed accordingly. | 6 |

Page 1 of 2

| **Section/topic** | **#** | **Checklist item** | **Reported on page #** |
| --- | --- | --- | --- |
| Risk of bias across studies | 15 | Publication bias was assessed using Eggers’s statistical test for each study. | 6 |
| Additional analyses | 16 | Sensitivity or subgroup analyses, meta-regression was done accordingly. | 6 |
| **RESULTS** | | |  |
| Study selection | 17 | 38 studies were included and summarized as PRISMA flow diagram | 6 |
| Study characteristics | 18 | It was presented in table in the amin document. | 6 |
| Risk of bias within studies | 19 | Risk of bias were detected in two factors written in the main document and in the figure list. | 12 |
| Results of individual studies | 20 | It was presented in the main document in the figure list accordingly. | 7 |
| Synthesis of results | 21 | Present results of each meta-analysis done, including confidence intervals and measures of consistency. | 9-12 |
| Risk of bias across studies | 22 | Present results of any assessment of risk of bias across studies (see Item 15). |  |
| Additional analysis | 23 | Give results of additional analyses, if done (e.g., sensitivity or subgroup analyses, meta-regression [see Item 16]). |  |
| **DISCUSSION** | | |  |
| Summary of evidence | 24 | Discussed accordingly. | 13-15 |
| Limitations | 25 | Written in the main document. | 15 |
| Conclusions | 26 | Written in the main document. | 15 |
| **FUNDING** | | |  |
| Funding | 27 | No fund for this study. | 16 |

*From:*  Moher D, Liberati A, Tetzlaff J, Altman DG, The PRISMA Group (2009). Preferred Reporting Items for Systematic Reviews and Meta-Analyses: The PRISMA Statement. PLoS Med 6(7): e1000097. doi:10.1371/journal.pmed1000097

For more information, visit: **www.prisma-statement.org**.
