# Supplementary material for: Prevalence of hypertension and its determinants in Ethiopia: A systematic review and meta-analysis
Source: PLoS One. 2020 Dec 31;15(12):e0244642. doi: 10.1371/journal.pone.0244642 (PMC7774863; doi:10.1371/journal.pone.0244642)
Supplement: Attachment — Submitted filename: Review_comments.docx [file pone.0244642.s012.docx]

General comment

This systematic review and meta-analysis is interesting in view of a rising burden of hypertension in LMICs and increasing number of publications. The analysis was handled very well and results presented appropriately. However, the written part especially in the introduction and discussions is rather sloppy. A lot of this is due to language difficulties which I’m sure can be addressed by hiring a native English speaker to edit.

I have specific comments for each section as follows:

**Introduction**

Apart from the common grammatical errors; the presentation of the literature on the burden needs to be re-organized in paragraphs into four main themes.

1-The Big picture (Burden of hypertension)

2- The specific problem (Literature about hypertension systematic reviews in Ethiopia and what the burden is)

3- The GAP (need to show the missing information in previous systematic reviews) and how this one fills the gap.

4- How this review fills the gap. One obvious reason is updating the previous reviews (I found 2 of them all published in 2015) and also state what you do to handle the review differently.

In the current form the is a mix of literature and sometimes contradicting information from two sources is posted but not explained example

For example the articles cited below speak about the same thing but are not brought together in a coherent way.

“Approximately 970 million people worldwide have high blood pressure and 1.56 billion adults will be living with hypertension by 2025 (3)”

“Globally around 1.13 billion people live with hypertension, which is two-thirds of them from low and middle-income countries (5).”

The statement that no systematic reviews were available to the knowledge of the authors is not true. I came across two systematic reviews on hypertension in Ethiopia and no mention of these were made apart from one that appeared in the discussion. The authors need to be honest about the scientific premise and show what gaps were in the previous review to warrant their review. See below the two reviews I came across

1-Kibret, K. T., & Mesfin, Y. M. (2015). Prevalence of hypertension in Ethiopia: a systematic meta-analysis. Public Health Reviews, 36(1), 14

2- Molla, M. (2015). Systematic reviews of prevalence and associated factors of hypertension in Ethiopia: finding the evidence. Sci J Public Health, 3(4), 514-9.

**Methods**

Just checking on the sensitivity of the search criteria: a synonym for high blood pressure “raised blood pressure” should have been indicated in the search terms. It is possible some articles could have been missed if they only used this terminology.

Inclusion criteria

Why did you choose 2000 as the earliest year to limit your search? Please give the rationale for selecting articles from this year.

Quality assessment

The statement below is confusing. Please clarify

“Based on the score of the quality assessment tool the highest score from nine questions declared low risk of bias” this is not clear. This would mean the highest score had the minimum risk yet the proceeding statement says the opposite

**Results**

You are silent about grey literature in your reporting yet you did mention an attempt to search for this in the methods section. Pease clarify if any grey literature was found and how it was handled.

**Discussion**

In the discussion section, a good attempt is made to interpret the findings but the language makes it rather sloppy. Need to hire the services of a native English speaker for language edits.

The comment in the limitation section about social desirability of the study is erroneous. The phrase social desirability bias is not appropriately used. I do not think cross sectional studies lead to social desirability. This could as well happen in longitudinal studies.

“Social desirability bias refers to the tendency of research subjects to give socially desirable responses instead of choosing responses that are reflective of their true feelings. The bias in responses due to this personality trait becomes a major issue when the scope of the study involves socially sensitive issues such as politics, religion, and environment, or personal issues such as drug use, cheating, and smoking. This is usually resolved by use of a well‐trained interviewer or collection of data through methods that do not require presence involvement of an interviewer can help avoid this bias to some extent. Properly identified options to questions vulnerable to social desirability effect is another means of tackling this issue”
