# Supplementary material for: Prevalence of hypertension and its determinants in Ethiopia: A systematic review and meta-analysis
Source: PLoS One. 2020 Dec 31;15(12):e0244642. doi: 10.1371/journal.pone.0244642 (PMC7774863; doi:10.1371/journal.pone.0244642)
Supplement: Attachment — Submitted filename: Response to Reviewers.docx [file pone.0244642.s014.docx]

# **Response to Reviewers’**

# **Prevalence and determinants of hypertension in Ethiopia: Systematic review and meta-analysis**

Sofonyas Abebaw Tiruneh^1^, Yeaynmarnesh Asmare Bukayaw^2^, Seblewongel Tigabu Yigizaw^2^, Dessie Abebaw Angaw^2^

The authors, extend great thanks for the editors and reviewers as the stand of this review. The comments raised by the reviewers are vital and defiantly it will improve the quality of the manuscript.

**Stay Safe!!!**

The Authors.

Reviewer #2: The language edits are not satisfactory. I doubt if the authors sought services of a native English speaker. I noted several grammatical errors. It appears to me more errors were introduced during the revisions. I have cited some of them here but this is not an exhaustive list. There will be need for a thorough check. I strongly recommend hiring a language editor.

Introduction
Lines 58-60- According to the World Health Organization (WHO) report in 2019, globally more than 1.13 billion people live with hypertension, of this two-thirds of them from Low and Middle-Income Countries (LMICs)


Lines 63-64. According to a systematic study, the overall pooled prevalence of hypertension in Africa raised from 19.7% in 1990, 27.4% in 2000 and 30.8% in 2010 [5].

Line 65: people live with hypertension and it will be projected 3 out of 4 people by the end of 2025

Lines 76-77: increment of non-communicable diseases will lead to greater dependency and rise the costs of health care for patients and their families unless public health interventions.

Line 82: Besides, more than half of the studies published after the previous study conducted.

Discussion
Line 305: Non-communicable disease was a double burden of public health problem in developing countries

Line 330: This finding was consistence with a study conducted in Nepal

We noted all the concerns and we have tried to our maximum effort to improve the quality of English writing.

We also invited the English exert river in our institution.
