# Supplementary material for: Prevalence of hypertension and its determinants in Ethiopia: A systematic review and meta-analysis
Source: PLoS One. 2020 Dec 31;15(12):e0244642. doi: 10.1371/journal.pone.0244642 (PMC7774863; doi:10.1371/journal.pone.0244642)
Supplement: Attachment — Submitted filename: Response to Reviewers.docx [file pone.0244642.s015.docx]

# **Response to Reviewers’**

# **Prevalence and determinants of hypertension in Ethiopia: Systematic review and meta-analysis**

Sofonyas Abebaw Tiruneh^1^, Yeaynmarnesh Asmare Bukayaw^2^, Seblewongel Tigabu Yigizaw^2^, Dessie Abebaw Angaw^2^

**Editors request**

The reviewers feel that the editorial changes were not satisfactory to merit publication. Particularly, the authors should pay particular attention to each and every sentence to be sure it has been properly proofed and vetted by a native English speaker for correct grammar and syntax. Without these changes we will be unable to publish this work that has some merit according to the reviewers and editorial staff. Please make these changes at your earliest convenience and resubmit.

**Authors response**

The authors, extend great thanks for the editors and reviewers as the stand of this review. The comments raised by the editors are valuable and defiantly it will improve the quality of the manuscript. We have addressed the English and grammar problems as per the editor request.

Since all authors are from low-income country, we cannot able to proofread the manuscript by fluent in English by payment especially native by English. But we have tried to improve the grammar and punctuation problems through read again and again. Besides, we had invited the manuscript for senior English editors at the University of Gondar and Debre Tabor University, Ethiopia. Those two English experts Lectures review our manuscript by free cost.

We hope that the English write up problem improves and will meet the minimum publication criteria of PLOS ONE journal. We look forwarding your excellent comments as usual.

The authors,
