# Supplementary material for: Prevalence of hypertension and its determinants in Ethiopia: A systematic review and meta-analysis
Source: PLoS One. 2020 Dec 31;15(12):e0244642. doi: 10.1371/journal.pone.0244642 (PMC7774863; doi:10.1371/journal.pone.0244642)
Supplement: Attachment — Submitted filename: Response Letter to Reviewer.R4.docx [file pone.0244642.s016.docx]

**Response Letter to editors and reviewers**

# Prevalence of hypertension and its determinants in Ethiopia: A systematic review and meta-analysis

Sofonyas Abebaw Tiruneh^1*^, Yeaynmarnesh Asmare Bukayaw^2^, Seblewongel Tigabu Yigizaw^2^, Dessie Abebaw Angaw^2^

We authors appreciate to the editors and reviewers for this manuscript as the position fourth review. The comments raised by the editors and reviewers are vital and defiantly it will improve the quality of the manuscript. We have addressed all the issues raised by the editors and reviewer's and believed that the revised version of the manuscript is satisfactory and will meet the minimum journal publication requirements. We have updated the manuscript accordingly.

**Stay Safe!!!**

The Authors.

Additional Editor Comments (if provided):

The scientific merit has certainly been met, however, the language simply is illegible by current English standards. This will be the authors final chance to amend the paper so that all grammatical mistakes are corrected and the paper flows in a smooth and readable fashion. For example the second sentence of the abstract reads, "A comprehensive electronic databases". There is a lack of subject/verb agreement, and this phrase is uninterpretable by the reader. Please make this final effort to correct the paper, and we look forward to a thoroughly reviewed next draft. Thank you
for your attention to these details.

*Noted: Thank you! We have made our final attempt as our maximum effort.*

**Review Comments to the Author**

Reviewer #1: I very much appreciate the efforts to address all my comments. However,

1. You probably did not get my comment on the introduction, I would suggest you simply cancel the first sentence of the introduction, it is controversial and adds nothing.

2. The language edits are really far below what is acceptable. My suggestion would be that you have the paper read by a scientific writer.

*Noted thank you, We appreciate your comment and We had corrected in the recent version of the manuscript*
